# Occurrence rate and risk factors for acute kidney injury after lung transplantation: a systematic review and meta-analysis

**DOI:** 10.7717/peerj.18364

**Published:** 2025-02-21

**Authors:** Nuan Lee, Haoxing Ying

**Affiliations:** 1 The Second Clinical Medical College, Wenzhou Medical University, Wenzhou, Zhejiang, China; 2 Medical College, Xijing University, Xi’an, Shanxi, China

**Keywords:** Lung transplantation, Acute kidney injury, Systematic review, Meta-analysis, Risk factors, Case-control studies

## Abstract

**Background:**

Compared with other solid organ transplantation, the morbidity rate of acute kidney injury is higher in lung transplantation. Our research was designed to examine the occurrence rate and risk factors for acute kidney injury after lung transplantation through a systematic review and meta-analysis.

**Methodology:**

We conducted a database search for case-control studies and cohort studies on the occurrence rate and risk factors for acute kidney injury after lung transplantation up to August 19, 2023. Stata 15.0 was used for data analysis.

**Results:**

Nineteen case-control or cohort studies were included, involving 1,755 cases of acute kidney injury after lung transplantation and 1,404 cases of non-acute kidney injury after lung transplantation. Based on the meta-analysis, the risk factors for acute kidney injury after lung transplantation included pulmonary fibrosis (OR, 1.34; CI [1.09–1.65]), hypertension (OR, 1.30; CI [1.07–1.58]), pre-op mechanical ventilation (OR, 3.30; CI [1.84–5.90]), pre-op extracorporeal membrane oxygenation (OR, 3.70; CI [2.51–5.45]), double lung transplantation (OR, 1.91; CI [1.45–2.53]), cardiopulmonary bypass support (OR, 1.82; CI [1.38–2.40]), cardiovascular events (OR, 1.50; CI [1.15–1.96]), intra-op hypotension (OR, 2.70; CI [1.42–5.14]), post-op extracorporeal membrane oxygenation (OR, 1.90; CI [1.20–3.01]), sepsis (OR, 3.20; CI [2.16–4.73]), dialysis (OR, 12.79; CI [6.11–26.8]).

**Conclusions:**

Based on the existing evidence, clinical professionals can implement early detection, diagnosis and treatment of patients with acute kidney injury after lung transplantation, to improve the quality of life of these patients.

## Introduction

Lung transplantation (LT) is known as the last treatment regimen for individuals with end-stage lung diseases (idiopathic pulmonary fibrosis, cystic pulmonary fibrosis, occupational lung diseases, chronic obstructive pulmonary disease, pulmonary hypertension, *etc*.) to prolong survival, improve quality of life and even to be cured (Chambers et al., 2017; Tabarelli et al., 2016). In recent years, lung transplantation has been increasingly performed globally, with over 4,000 successful procedures conducted annually (Chambers et al., 2018). With the rapid development of transplantation technique and accumulation of experience, the median survival following LT has improved from 5.6 years a decade ago to the recent 6.7 years, which is still significantly lower than that of other solid organ transplantation (SOT) (Chambers et al., 2021). With the advancement of surgical techniques and the improvement of perioperative management, the short-term as well as long-term survival rates in LT recipients have been significantly improved, but complications after LT are still the main factors affecting the survival rate of recipients (Chambers et al., 2017; Lyu & Zamora, 2009).

In the Kidney Disease Improving Global Outcome (KDIGO) clinical practice guidelines, acute kidney injury (AKI) is defined as any of the following: increase in serum creatinine (SCr) by ≥0.3 mg/dl (≥26.5 µmol/l) within 48 h; or an increase in serum creatinine to ≥1.5 times baseline, which is known or presumed to have occurred within the preceding 7 days; or a urine volume <0.5 ml/kg/h for 6 hours (Khwaja, 2012). AKI is caused by various pathological conditions like renal hypoperfusion, sepsis, exposure to nephrotoxic agents, or major renal surgery (Ronco, Bellomo & Kellum, 2019). Due to comprehensive factors such as lung ischemia-reperfusion injury, rapid hemodynamic changes and surgical trauma, LT patients are prone to AKI, with the occurrence rate of about 39.0% to 74.5% (Jacques et al., 2012). AKI after LT is related to higher mortality and morbidity, and it also has a relationship with primary graft dysfunction (PGD) and longer ICU stays (Arnaoutakis et al., 2011; Nguyen et al., 2017; Wehbe et al., 2012). Previous studies have indicated that renal insufficiency is a common complication after LT, and the morbidity and mortality rates of AKI after LT are still high (Rocha et al., 2005). Compared with liver transplantation and other SOT, the morbidity rate of AKI is high, and the use of continuous renal replacement therapy (CRRT) is more common in LT recipients (Chang, Chan & Patterson, 2023; Lertjitbanjong et al., 2019; Thongprayoon et al., 2019).

As a result, it is of positive significance to study the risk factors (RFs) for AKI after LT, but there are few studies on the occurrence rate of AKI after LT, and the RFs remain controversial. The difference is mainly due to complex pathophysiological mechanisms. There are significant differences in physiological status and basic diseases between different patients, which may lead to different responses of patients to the same risk factors, increasing the complexity of risk factors; after lung transplantation, AKI may be caused by interactions between multiple factors, including hemodynamic changes during surgery, the impact of immunosuppressive therapy, infection, and drug-related side effects. Hence, it is difficult to fully explain the occurrence of AKI with a single factor (Arnaoutakis et al., 2011; Jacques et al., 2012). We, therefore, conducted this study to explore the RFs for AKI after LT through a systematic review and meta-analysis to resolve such controversy, hoping to provide a reference for further development of targeted prevention strategies for AKI after LT and improve the quality of life and the prognosis in LT recipients.

## Materials & Methods

The study protocol was developed in accordance with the Preferred Reporting Items for Systematic Review and Meta-Analysis Protocols (PRISMA-P) guidelines, and the review was conducted according to the PRISMA guidelines (Liberati et al., 2009).

### PROSPERO registration

The protocol ‘A Meta-analysis of Risk factors and Incidence of Acute Renal Injury after Lung Transplantation’ has been registered on PROSPERO with registration number PROSPERO CRD42024457767.

### Literature search

CNKI, PubMed, Embase, Cochrane Library, and Web of Science were searched for case-control and cohort studies on the occurrence rate of and RFs for AKI in LT patients up to August 19, 2023. Subject terms and free words were used for literature retrieval, including lung transplantation, acute kidney injury, and RFs. The specific retrieval strategies are provided in Supplemental Information 1.

### Eligibility criteria

Inclusion criteria: The studies must have been case-control studies or cohort studies. The participants in the studies were adults receiving LT, and the exposure factor was RF for AKI after LT. The studies must have reported the occurrence of AKI after LT as primary outcome, either through univariate or multivariate analysis, or both; however, when the same study included both the univariate and the multivariate analyses, the multivariate analysis results were preferred, and the multivariate-adjusted risk values were extracted. If only the univariate analysis was included, the univariate analysis results were extracted. No restriction was imposed on language.

Exclusion criteria: meeting abstracts, meta-analyses, protocols, letters, overlapping publications, systematic reviews, studies in which the full texts were unavailable, studies in which the data were unavailable, and animal experiments were excluded.

### Literature screening and data extraction

Two independent reviewers screened the literature and extracted the data separately. Through reading the titles and abstracts of the studies, the two reviewers directly included the literature on which they agreed with each other for full-text screening; disagreements were resolved through discussion or consultation with the third investigator, Xinran Yang. The full texts of potentially relevant studies were downloaded and read to select eligible studies in strict accordance with the eligibility criteria. The following data were extracted, covering the first author’s name, gender, country, study design, sample size, publication year, and age. The extracted data were cross-checked to ensure data consistency.

### Quality assessment

We assessed all the case-control studies and cohort studies using the Newcastle-Ottawa Scale (NOS) (Stang, 2010), including study population selection (four points), comparability between groups (two points), and exposure factors or outcome measures (three points). The scale has a total score of 9, with a score of ≤ 4 being considered as low quality (Jacques et al., 2012; Ronco, Bellomo & Kellum, 2019), as moderate quality, and ≥ 7 as high quality. Disagreements, if any, were discussed between the same two researchers or addressed through consultation with a third researcher.

### Statistical analysis

Stata 15.0 was adopted for statistical analysis of the data. We used the DL method for pooling the effects, analyzing between-study variances, and calculating the 95% confidence intervals (CI). We use odds ratio (OR) values to describe the strength of the association between risk factors and AKI after LT in univariate analysis, and analyzed studies that used hazard ratio (HR) (Atchade et al., 2020; Balci et al., 2017; Du et al., 2021; Fidalgo et al., 2014; Greite et al., 2022; Ishikawa, Griesdale & Lohser, 2014; Jacques et al., 2012; Kim et al., 2021; Liu et al., 2021; Lu et al., 2023; Rocha et al., 2005; Sang et al., 2021; Scaravilli et al., 2022; Xue et al., 2014; Zhang Shuai & Jingqing, 2020) and odds ratio (OR) (Chan et al., 2023; Jing et al., 2021; Kim et al., 2021; Toyoda et al., 2023) respectively while conducting multivariate analysis. As for heterogeneity test (Q test), for *I*^2^ > 50%, the one-by-one elimination method was adopted for sensitivity analysis of the literature, and Egger’s test was employed for publication bias analysis, with a significant level of *α* = 0.05. A *P*-value < 0.05 implied that the difference was of statistical significance.

## Results

### Literature retrieval results

**Figure 1:**
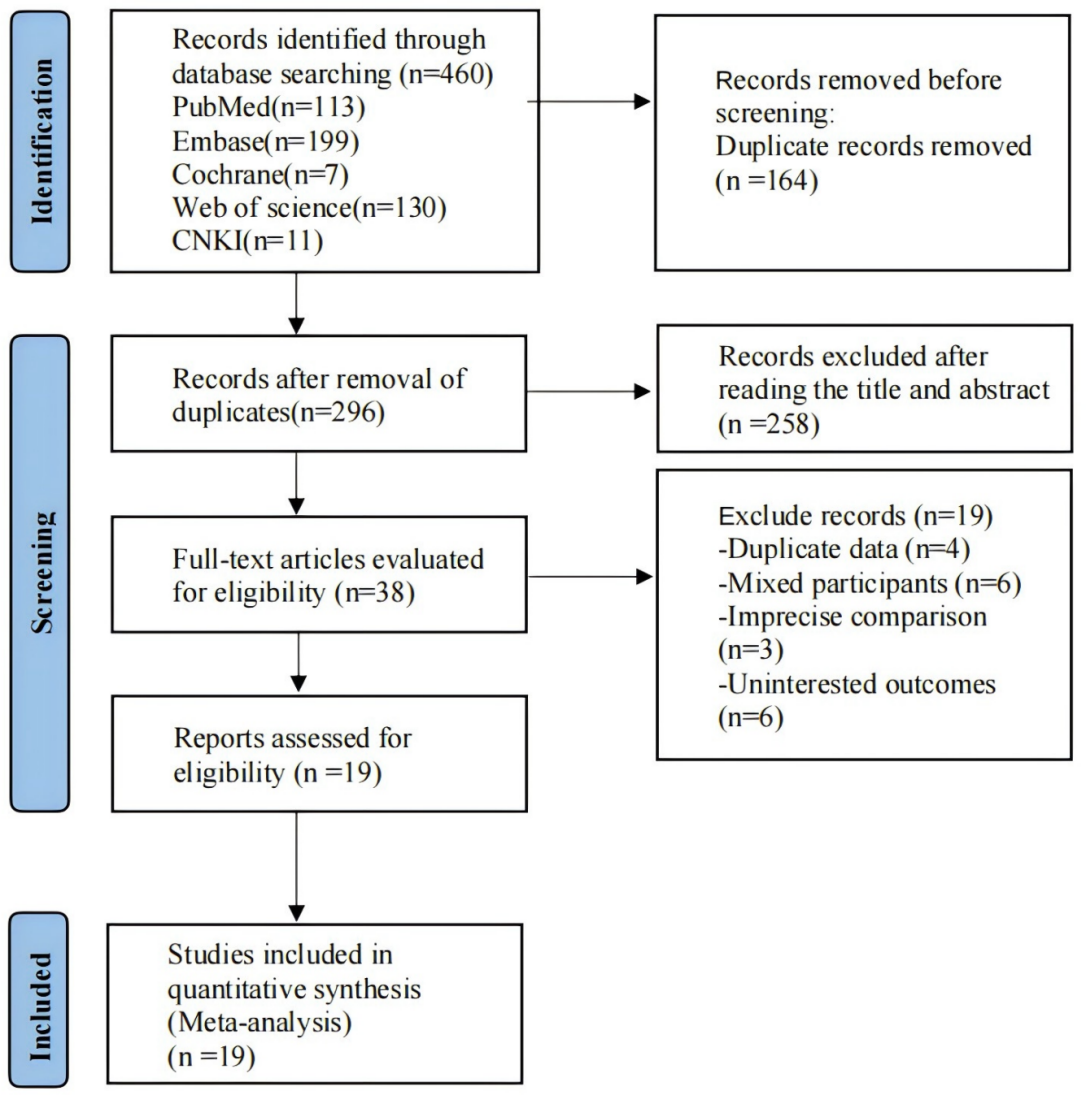
Literature screening.

A total of 496 records were retrieved. There were 296 records identified after removing duplications, and 38 records identified after reading the titles and abstracts. By reading the full texts of potentially relevant studies, we finally included 19 studies, consisting of one Chinese publication (Zhang Shuai & Jingqing, 2020), and 18 English publications. The specific search flow chart is provided in Fig. 1.

### Basic characteristics of the included literature

All the included nineteen studies were case-control or cohort studies (Atchade et al., 2020; Balci et al., 2017; Chan et al., 2023; Du et al., 2021; Fidalgo et al., 2014; Greite et al., 2022; Ishikawa, Griesdale & Lohser, 2014; Jacques et al., 2012; Jing et al., 2021; Kim et al., 2021; Liu et al., 2021; Lu et al., 2023; Rocha et al., 2005; Sang et al., 2021; Scaravilli et al., 2022; Sikma et al., 2017; Toyoda et al., 2023; Viera, 2008; Xue et al., 2014; Zhang Shuai & Jingqing, 2020), including 1,755 LT patients with AKI and 1,404 LT patients without AKI. The age of patients had a range from 22 to 67 years old. Table 1 displays the specific characteristics of the included studies. The included studies had a score of seven to eight points using the NOS scale, with overall high quality, and the specific quality evaluation results are provided in Table 2.

**Table 1:** The specific characteristics of the included studies.

Study	Country	**Language**	**Study design**	Sample size		Gender (M/F)	Age (years)		Diagnostic criteria for AKI
				S	NS		S	NS	
Atchade et al. (2020)	France	English	Cohort study	46	48	60/34	50–59	47–67	KDIGO
Balci et al. (2017)	Turkey	English	Case-control study	16	14	19/11	46.1	39.2	AKIN
Chan et al. (2023)	America	English	Case-control study	369	385	441/313	51–67		KDIGO
Du et al. (2021)	China	English	Case-control study	110	26	117/19	54–64	54–66	KDIGO
Fidalgo et al. (2014)	Canada	English	Case-control study	306	139	276/169	39–60	42–60	KDIGO
Greite et al. (2022)	Germany	English	Cohort study	10	10	10/10	49.9	48.7	KDIGO
Ishikawa, Griesdale & Lohser (2014)	Japan	English	Case-control study	27	23	31/19	49	56	RIFLE
Jacques et al. (2012)	Canada	English	Case-control study	67	107	83/91	46	46	RIFLE
Jing et al. (2021)	China	English	Case-control study	137	54	159/32	54–62	49–62	KDIGO
Kim et al. (2021)	Korea	English	Case-control study	59	89	89/59	55.24	53.13	KDIGO
Liu et al. (2021)	China	English	Case-control study	19	6	21/4	55.6	61.8	KDIGO
Lu et al. (2023)	China	English	Case-control study	21	10	27/4	58.4	59.9	KDIGO
Rocha et al. (2005)	America	English	Case-control study	166	130	153/143	44	51	RIFLE
Sikma et al. (2017)	Netherlands	English	Case-control study	85	87	83/89	45	47	KDIGO
Sang et al. (2021)	China	English	Case-control study	67	81	125/23	57.1	54.5	KDIGO
Scaravilli et al. (2022)	Italy	English	Case-control study	50	31	40/41	22–38	24–37	KDIGO
Toyoda et al. (2023)	America	English	Case-control study	110	96	114/92	56.6	58.4	KDIGO
Xue et al. (2014)	China	Chinese	Case-control study	47	41	67/21	53.45	51.88	AKIN
Zhang Shuai & Jingqing (2020)	China	English	Case-control study	43	27	61/9	55–64	58–67	KDIGO

**Notes.**

NS: Lung transplantation without acute kidney injury; S: Lung transplantation with acute kidney injury; KDIGO: the Improving Global Outcomes Acute Kidney Injury Criteria, people who meet one of the following conditions are diagnosed with AKI: ① Scr increases by over 26.5 μmol/L (0.3 mg/dl) within 48 hours; ② Scr rises more than 1.5 times baseline within seven days; ③ Urine volume < 0.5 ml/(kg h), which last for over 6 hours; AKIN: the Acute Kidney Injury Net classification, an abrupt (within 48 hours) reduction in kidney function currently defined as an absolute increase in serum creatinine of more than or equal to 0.3 mg/dl (>26.4 μmol/l), a percentage increase in serum creatinine of more than or equal to 50% (1.5-fold from baseline), or a reduction in urine output (documented oliguria of less than 0.5 ml/kg per hour for more than six hours); RIFLE: The risk, injury, failure, loss, endstage renal disease criteria, Scr rises more than 1.5 times baseline, or GFR reduces by over 25%, or urine volume < 0.5 ml/(kg h) for over 6 hours.

**Table 2:** The NOS quality evaluation. Each asterisk (*) represents a score in the Newcastle-Ottawa Scale (NOS), a tool used to assess the quality of non-randomized controlled studies. High, NOS score > 7 points; low, NOS score < 6 points; moderate, 6–7 points.

Study	Definition adequate	Represen- tativeness of the cases	Definition of Controls	Comparability of cases and controls on the basis of the design or analysis	Ascertainment of exposure	Same method of ascertainment for cases and controls	Non response	Total scores
Atchade et al. (2020)	*	*	*	**	*	*	*	8
Balci et al. (2017)	*	*	*	**	*	*	*	8
Chan et al. (2023)	*	*	*	**	*	*	*	8
Du et al. (2021)	*	*	*	**	*	*	*	8
Fidalgo et al. (2014)	*	*	*	**	*	*	*	8
Greite et al. (2022)	*	*	*	**	*	*	*	8
Ishikawa, Griesdale & Lohser (2014)	*	*	*	**	*	*	*	8
Jacques et al. (2012)	*	*	*	**	*	*	*	8
Jing et al. (2021)	*	*	*	**	*	*	*	8
Kim et al. (2021)	*	*	*	**	*	*	*	8
Liu et al. (2021)	*	*	*	**	*	*	*	8
Lu et al. (2023)	*	*	*	*	*	*	*	7
Rocha et al. (2005)	*	*	*	*	*	*	*	7
Sikma et al. (2017)	*	*	*	**	*	*	*	8
Sang et al. (2021)	*	*	*	**	*	*	*	8
Scaravilli et al. (2022)	*	*	*	*	*	*	*	7
Toyoda et al. (2023)	*	*	*	**	*	*	*	8
Xue et al. (2014)	*	*	*	**	*	*	*	8
Zhang Shuai & Jingqing (2020)	*	*	*	*	*	*	*	7

### Publication bias

Egger’s test was adopted to assess possible publication bias for each RF. When interpreting meta-analysis results, we fully considered the possibility of publication bias and its impact. When there was publication bias for variables reported in only two studies, and it is difficult to prove that the two studies are of extremely high quality, consistency, and comparability, they cannot provide valuable information for meta-analysis and should not be included. In the univariate analysis, pulmonary fibrosis (PF), hypertension, pre-op mechanical ventilation (MV), pre-op extracorporeal membrane oxygenation (ECMO), double lung transplantation (LT), cardiopulmonary bypass (CPB) support, cardiovascular events, intra-op hypotension, post-op ECMO, dialysis, lung allocation score (LAS), operating time, transfusion red blood cell (RBC), transfusion plasma, duration of ECMO support and duration of MV had no publication bias (*P*-value > 0.05), sepsis had publication bias (*P*-value < 0.05), as shown in Table 3. The result of sepsis was interpreted with caution, and this limitation would be described in the Discussion section. In the multivariate analysis, double LT didn’t exist publication bias, as shown in Table 4.

**Table 3:** The univariate analysis results.

Risk factors	No. of study	Heterogeneity		OR/SMD (95% CI)	*P*	*P* of Egger
		*I*^2^ (%)	*P*			
Binary variables						
Male	19	43.6	0.023	0.91 (0.78, 1.06)	0.206	0.319
Female	19	43.6	0.023	1.10 (0.95, 1.28)	0.206	0.319
Smoking history	4	55.7	0.079	0.71 (0.37, 1.38)	0.314	0.166
Previous chest surgery	3	45.7	0.159	1.07 (0.76, 1.51)	0.686	0.680
Donor Men	2	0	0.372	0.59 (0.37, 0.92)	0.022	<0.001
Pulmonary fibrosis	14	30.2	0.135	1.34 (1.09, 1.65)	0.006	0.912
Chronic obstructive pulmonary disease	12	51.7	0.019	0.78 (0.54, 1.11)	0.164	0.015
Interstitial lung disease	6	47.5	0.090	1.03 (0.74, 1.42)	0.875	0.703
Pulmonary hypertension	6	0	0.552	1.19 (0.74, 1.89)	0.473	0.239
Alpha-1-anti-trrysine deficiency	3	27.3	0.253	0.69 (0.33, 1.45)	0.328	0.623
Bronchiectasis	6	43.2	0.117	1.04 (0.59, 1.82)	0.902	0.679
Bronchiolitis obliterans	2	0	0.320	1.22 (0.41, 3.59)	0.722	<0.001
Hypertension	15	0	0.467	1.30 (1.07, 1.58)	0.007	0.514
Diabetes mellitus	18	0	0.633	1.18 (0.97, 1.45)	0.104	0.284
Dyslipidemia	5	0	0.887	1.58 (0.95, 2.63)	0.077	0.200
Chronic kidney failure	5	47.7	0.105	1.05 (0.62, 1.77)	0.862	0.303
Pulmonary hypertension	4	0	0.606	1.33 (0.90, 1.96)	0.149	0.017
Peripheral vascular disease	2	0	0.732	1.06 (0.24, 4.61)	0.038	<0.001
Cerebrovascular disease	2	46.6	0.171	0.48 (0.08, 2.85)	0.032	<0.001
Coronary artery disease	5	0	0.416	1.23 (0.85, 1.77)	0.269	0.163
Creatinine clearance > 90 mL min	2	38.3	0.203	1.49 (0.99, 2.24)	0.055	<0.001
Recipient CMV+	2	13	0.284	0.94 (0.67, 1.32)	0.724	<0.001
Pre-op MV	6	0	0.655	3.30 (1.84, 5.90)	<0.001	0.061
Pre-op ECMO	11	0	0.634	3.70 (2.51, 5.45)	<0.001	0.510
Double lung transplantation	15	53.3	0.008	1.91 (1.45, 2.53)	<0.001	0.949
Single lung transplantation	9	44.1	0.074	0.52 (0.42, 0.64)	<0.001	0.137
Intra-op ECMO support	13	39.8	0.068	1.19 (0.97, 1.46)	0.099	0.808
Cardiopulmonary bypass support	5	0	0.991	1.82 (1.38, 2.40)	<0.001	0.455
Cardiovascular events	4	3.4	0.376	1.50 (1.15, 1.96)	0.003	0.308
Blood cell transfusion	2	7.7	0.298	1.93 (1.00, 3.75)	0.051	<0.001
Intra-op hypotension	3	30.7	0.236	2.70 (1.42, 5.14)	0.003	0.823
Aprotinin use	3	52.3	0.123	1.67 (0.82, 3.38)	0.157	0.040
Post-op ECMO	7	0	0.773	1.90 (1.20, 3.01)	0.006	0.681
Reoperation	2	0	0.550	1.89 (0.91, 3.94)	0.089	<0.001
Sepsis	6	0	0.674	3.20 (2.16, 4.73)	<0.001	0.012
Stage 3 primary graft dysfunction	3	76.8	0.013	2.29 (0.86, 6.10)	0.096	0.035
Primary graft dysfunction	2	44.4	0.180	5.59 (2.62, 11.93)	<0.001	<0.001
Multiple organ dysfunction during ICU	2	0	0.422	11.11 (4.17, 29.61)	<0.001	<0.001
pH < 7.20 on ICU admission	2	46.8	0.170	0.87 (0.39, 1.90)	0.720	<0.001
Lactate > 3 mmol L	2	18.3	0.269	2.52 (1.50, 4.25)	<0.001	<0.001
Mechanical ventilation > 3 d	2	0	0.344	2.68 (1.53, 4.69)	0.001	<0.001
Dialysis	5	0	0.551	12.79 (6.11, 26.8)	<0.001	0.415
Repeat chest surgery	2	32.6	0.223	0.92 (0.40, 2.11)	0.846	<0.001
Pneumonia	2	0	0.734	4.04 (1.90, 8.61)	<0.001	<0.001
Ganciclovir	2	0	0.659	1.16 (0.61, 2.21)	0.654	<0.001
Basiliximab	2	0	0.816	1.02 (0.61, 1.70)	0.945	<0.001
Cyclosporine	4	73.2	0.011	0.73 (0.40, 1.32)	0.296	0.668
Supra-therapeutic whole-blood tacrolimus trough concentration	2	0	0.738	2.27 (1.16, 4.48)	0.017	<0.001
Tacrolimus	4	25.5	0.258	1.09 (0.81, 1.45)	0.572	0.680
Amphotericin B intravenous infused	2	39.5	0.199	2.88 (1.5, 5.54)	0.001	<0.001
Continuous variables						
Age, year	19	20.3	0.207	−0.04 (−0.11, 0.04)	0.319	0.861
BMI, kg/m^2^	14	24.1	0.194	−0.03 (−0.13, 0.07)	0.535	0.196
Donor Age, year	3	0	0.949	0.03 (−0.14, 0.21)	0.719	0.669
FEV_1_, %	2	0	0.554	−0.35 (−0.83, 0.12)	0.144	<0.001
Lung allocation score	4	0	0.394	0.34 (0.24, 0.45)	<0.001	0.866
Pre-op APACHE II score	4	76.7	0.005	0.19 (−0.26, 0.64)	0.407	0.482
SCr, mg/dl	13	86.9	<0.001	−0.05 (−0.32, 0.22)	0.715	0.325
GFR, mL/min	12	82.2	<0.001	0.21 (−0.02, 0.45)	0.068	0.013
ALT, IU/L	2	43.8	0.182	−0.05 (−0.37, 0.27)	0.755	<0.001
AST, IU/L	2	0	0.666	0.13 (−0.19, 0.45)	0.430	<0.001
WBC, ×109/L	2	0	0.884	0.2 (−0.04, 0.43)	0.108	<0.001
Platelets, ×109/L	2	0	0.439	−0.04 (−0.28, 0.2)	0.740	<0.001
Hemoglobin, g/L	5	61.6	0.034	−0.29 (−0.58, 0.00)	0.051	0.452
Total bilirubin, mg/dL	2	55.7	0.133	0.07 (−0.34, 0.47)	0.742	<0.001
BUN, mg/dL	2	0	0.349	0.03 (−0.21, 0.27)	0.818	<0.001
Operating time, min	13	67.1	<0.001	0.22 (0.03, 0.4)	0.023	0.365
Waitlist time, d	2	10.1	0.292	−0.02 (−0.16, 0.12)	0.767	<0.001
Mean arterial pressure, mmHg	2	0	0.758	−0.02 (−0.30, 0.27)	0.901	<0.001
Blood transfusions, ml	5	34.8	0.189	0.12 (−0.08, 0.32)	0.249	0.801
Transfusion RBC, ml	5	52.6	0.077	0.33 (0.08, 0.58)	0.011	0.676
Transfusion Plasma, ml	4	49	0.141	0.38 (0.12, 0.64)	0.004	0.065
Transfused platelets, ml	3	0	0.784	−0.15 (−0.45, 0.16)	0.346	<0.001
Total crystalloid, mL	2	0	0.527	−0.02 (−0.28, 0.25)	0.903	<0.001
Loss of blood, mL	6	56.4	0.043	0.16 (−0.12, 0.45)	0.264	0.916
Cardiopulmonary bypass, min	2	29.7	0.233	0.07 (−0.09, 0.24)	0.390	<0.001
Ischemia time, min	6	53	0.059	0.20 (0.00, 0.40)	0.050	0.475
Median TAC concentration, ng/mL	2	0	0.937	0.60 (0.34, 0.85)	<0.001	<0.001
Intraoperative fluid intake, ml	2	89.3	0.002	−0.12 (−1.54, 1.30)	0.868	<0.001
Intra-op Fluid balance, ml	3	63.9	0.063	0.39 (−0.10, 0.87)	0.117	0.059
Duration of ECMO support, d	4	0	0.758	0.36 (0.16, 0.57)	<0.001	0.132
Duration of MV, d	12	77.9	<0.001	0.47 (0.20, 0.74)	0.001	0.624
Day 1 SCr levels, mg/dL	2	25.6	0.246	0.77 (0.17, 1.37)	0.012	<0.001
Day 3 SCr levels, mg/dL	2	79.6	0.027	0.53 (−0.67, 1.73)	0.384	<0.001

**Table 4:** The multivariate analysis results.

Risk factors	No. of study	Heterogeneity		ES (Effect size, 95% CI)	*P*	*P* of Egger
		*I*^2^ (%)	*P*			
Age	4	12.8	0.329	0.01 (−0.02, 0.04)	0.477	0.188
BMI	2	0	0.462	−0.35 (−0.65, −0.05)	0.021	<0.001
Male	2	80	0.025	−0.35 (−1.53, 0.83)	0.565	<0.001
eGFR, mL/min	3	80.1	0.007	0.00 (−0.02, 0.02)	0.999	0.153
Pulmonary hypertension before surgery	2	76.1	0.410	0.24 (−1.05, 1.54)	0.711	<0.001
Diabetes	2	0	0.708	0.05 (−0.71, 0.80)	0.870	<0.001
Pulmonary hypertension	2	46.1	0.173	0.38 (−1.15, 1.91)	0.623	<0.001
Cystic fibrosis	2	0	0.975	0.77 (0.24, 1.30)	0.040	<0.001
Double lung transplantation	4	0	0.446	1.09 (0.59, 1.58)	<0.001	0.722
Cardiopulmonary bypass	2	0	0.564	0.00 (0.00, 0.01)	0.018	<0.001
Intra-op ECMO	4	0	0.876	0.09 (−0.43, 0.61)	0.742	0.526
Duration of mechanical ventilation	3	96.5	<0.001	0.17 (−2.79, 3.13)	0.909	0.638
Post-op ECMO	2	14.9	0.278	1.55 (0.15, 2.96)	0.030	<0.001
Mechanical ventilation >3 d	2	82.3	0.018	0.77 (−1.08, 2.62)	0.416	<0.001

### Occurrence rate of AKI after LT

Nineteen studies reported the morbidity rate of AKI after LT. Significant heterogeneity was found (*I*^2^ = 90.4%, *P* = 0.001). The meta-analysis showed that the occurrence rate of AKI after LT was 57% (ES = 57%; CI [51%–62%]). This occurrence rate of AKI after LT was estimated based on the current included studies instead of a natural occurrence rate, which should be interpreted with caution. Because the heterogeneity of this indicator was large, sensitivity analysis was conducted on the indicator using a one-by-one elimination method. The analysis results revealed small sensitivity, indicating that the analysis results were stable Egger’s test was conducted on this indicator to evaluate the publication bias, and a *P*-value = 0.997 was calculated, indicating a small possibility of publication bias for this indicator, as shown in Supplemental Information 2.

### Univariate meta-analysis results

#### Preoperative univariate analysis

**Figure 2:**
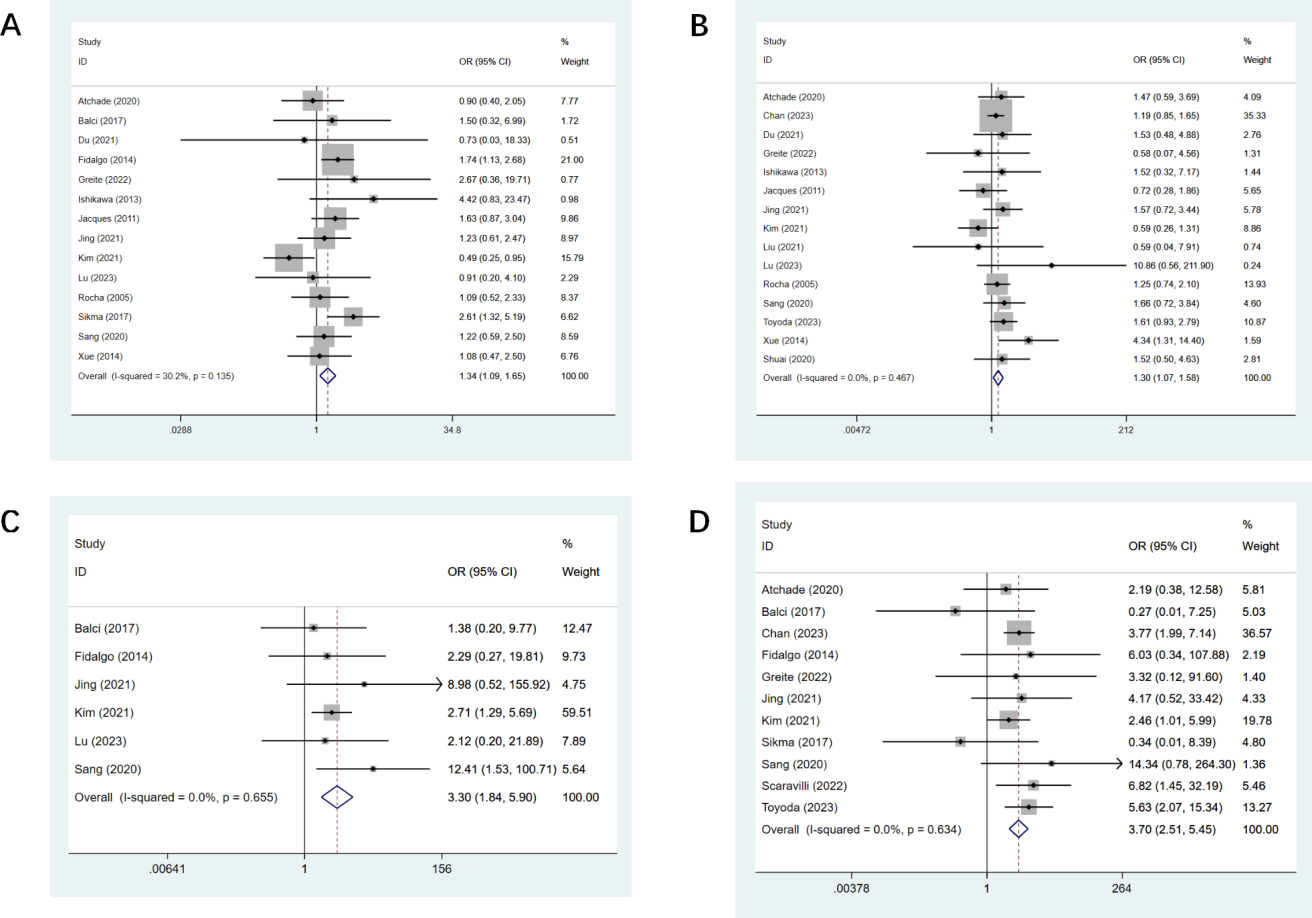
Forest plot showing the results for preoperative univariate analysis. (A) Pulmonary fibrosis; (B) hypertension; (C) pre-op mechanical ventilation; (D) pre-op ECMO. Studies: Atchade et al., 2020; Balci et al., 2017; Fidalgo et al., 2014; Jing et al., 2021; Kim et al., 2021; Lu et al., 2023; Sang et al., 2021; Greite et al., 2022; Ishikawa, Griesdale & Lohser, 2014; Jacques et al., 2012; Rocha et al., 2005; Chan et al., 2023; Du et al., 2021; Toyoda et al., 2023; Scaravilli et al., 2022; Sikma et al., 2017; Liu et al., 2021; Xue et al., 2014; Xue et al., 2014; Zhang Shuai & Jingqing, 2020.

PF was reported in 14 studies (Atchade et al., 2020; Balci et al., 2017; Du et al., 2021; Fidalgo et al., 2014; Greite et al., 2022; Ishikawa, Griesdale & Lohser, 2014; Jacques et al., 2012; Jing et al., 2021; Kim et al., 2021; Lu et al., 2023; Rocha et al., 2005; Sang et al., 2021; Sikma et al., 2017; Xue et al., 2014), and there was a statistical difference observed (OR, 1.34; CI [1.09–1.65]). The detailed effect sizes are outlined in Fig. 2A. Fifteen studies mentioned hypertension (Atchade et al., 2020; Du et al., 2021; Greite et al., 2022; Ishikawa, Griesdale & Lohser, 2014; Jacques et al., 2012; Jing et al., 2021; Kim et al., 2021; Liu et al., 2021; Lu et al., 2023; Rocha et al., 2005; Sang et al., 2021; Sikma et al., 2017; Toyoda et al., 2023; Viera, 2008; Xue et al., 2014; Zhang Shuai & Jingqing, 2020), and there was a statistical difference (OR, 1.30; CI [1.07–1.58], Fig. 2B). Six studies reported pre-op MV (Balci et al., 2017; Fidalgo et al., 2014; Jing et al., 2021; Kim et al., 2021; Lu et al., 2023; Sang et al., 2021), and a statistical difference was observed (OR, 3.30; CI [1.84–5.90], Fig. 2C). Eleven studies mentioned pre-op ECMO (Atchade et al., 2020; Balci et al., 2017; Chan et al., 2023; Fidalgo et al., 2014; Greite et al., 2022; Jing et al., 2021; Kim et al., 2021; Sang et al., 2021; Scaravilli et al., 2022; Sikma et al., 2017; Toyoda et al., 2023; Viera, 2008), and a statistical difference was found (OR, 3.70; CI [2.51–5.45], Fig. 2D). LAS was mentioned in four studies (Fidalgo et al., 2014; Scaravilli et al., 2022; Toyoda et al., 2023; Viera, 2008), and we found a statistical difference (OR, 0.34; CI [0.24–0.45]). All results of preoperative univariate analysis are provided in Table 3.

### Intraoperative univariate analysis

**Figure 3:**
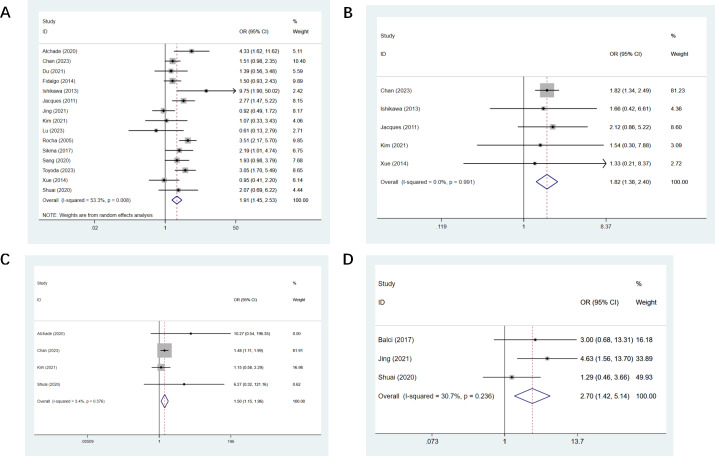
Forest plot showing the results for intraoperative univariate analysis. (A) Double lung transplantation; (B) cardiopulmonary bypass support; (C) cardiovascular events; (D) intra-op hypotension. Studies: Atchade et al., 2020; Balci et al., 2017; Fidalgo et al., 2014; Jing et al., 2021; Kim et al., 2021; Lu et al., 2023; Sang et al., 2021; Greite et al., 2022; Ishikawa, Griesdale & Lohser, 2014; Jacques et al., 2012; Rocha et al., 2005; Chan et al., 2023; Du et al., 2021; Toyoda et al., 2023; Scaravilli et al., 2022; Sikma et al., 2017; Liu et al., 2021; Xue et al., 2014; Xue et al., 2014; Zhang Shuai & Jingqing, 2020.

Fifteen studies reported double LT (Atchade et al., 2020; Du et al., 2021; Fidalgo et al., 2014; Ishikawa, Griesdale & Lohser, 2014; Jacques et al., 2012; Jing et al., 2021; Kim et al., 2021; Lu et al., 2023; Rocha et al., 2005; Sang et al., 2021; Sikma et al., 2017; Toyoda et al., 2023; Viera, 2008; Xue et al., 2014; Zhang Shuai & Jingqing, 2020), and a statistical difference was found (OR, 1.91; CI [1.45–2.53], Fig. 3A). Five studies mentioned CPB support (Ishikawa, Griesdale & Lohser, 2014; Jacques et al., 2012; Kim et al., 2021; Viera, 2008; Xue et al., 2014), we found a statistical difference (OR, 1.82; CI [1.38–2.40], Fig. 3B). Cardiovascular events were mentioned in four studies (Atchade et al., 2020; Kim et al., 2021; Viera, 2008; Zhang Shuai & Jingqing, 2020), a statistical difference was observed (OR, 1.50; CI [1.15–1.96], Fig. 3C). Three studies mentioned intra-op hypotension (Balci et al., 2017; Jing et al., 2021; Zhang Shuai & Jingqing, 2020), and the observed difference was of statistical significance (OR, 2.70; CI [1.42–5.14], Fig. 3D). Thirteen studies mentioned operating time with a statistical difference (OR, 0.22; CI [0.03–0.40]) (Balci et al., 2017; Du et al., 2021; Greite et al., 2022; Ishikawa, Griesdale & Lohser, 2014; Jing et al., 2021; Kim et al., 2021; Lu et al., 2023; Sang et al., 2021; Toyoda et al., 2023; Viera, 2008; Xue et al., 2014; Zhang Shuai & Jingqing, 2020). Five studies mentioned the amount of RBC transfusion (Greite et al., 2022; Kim et al., 2021; Sang et al., 2021; Scaravilli et al., 2022; Toyoda et al., 2023), and the observed difference was of statistical significance (OR, 0.33; CI [0.08–0.58]). Four studies mentioned the amount of plasma transfusion (Greite et al., 2022; Sang et al., 2021; Toyoda et al., 2023; Zhang Shuai & Jingqing, 2020), and the observed difference was of statistical significance (OR, 0.38; CI [0.12–0.64]). All results of intraoperative univariate analysis are presented in Table 3.

### Postoperative univariate analysis

**Figure 4:**
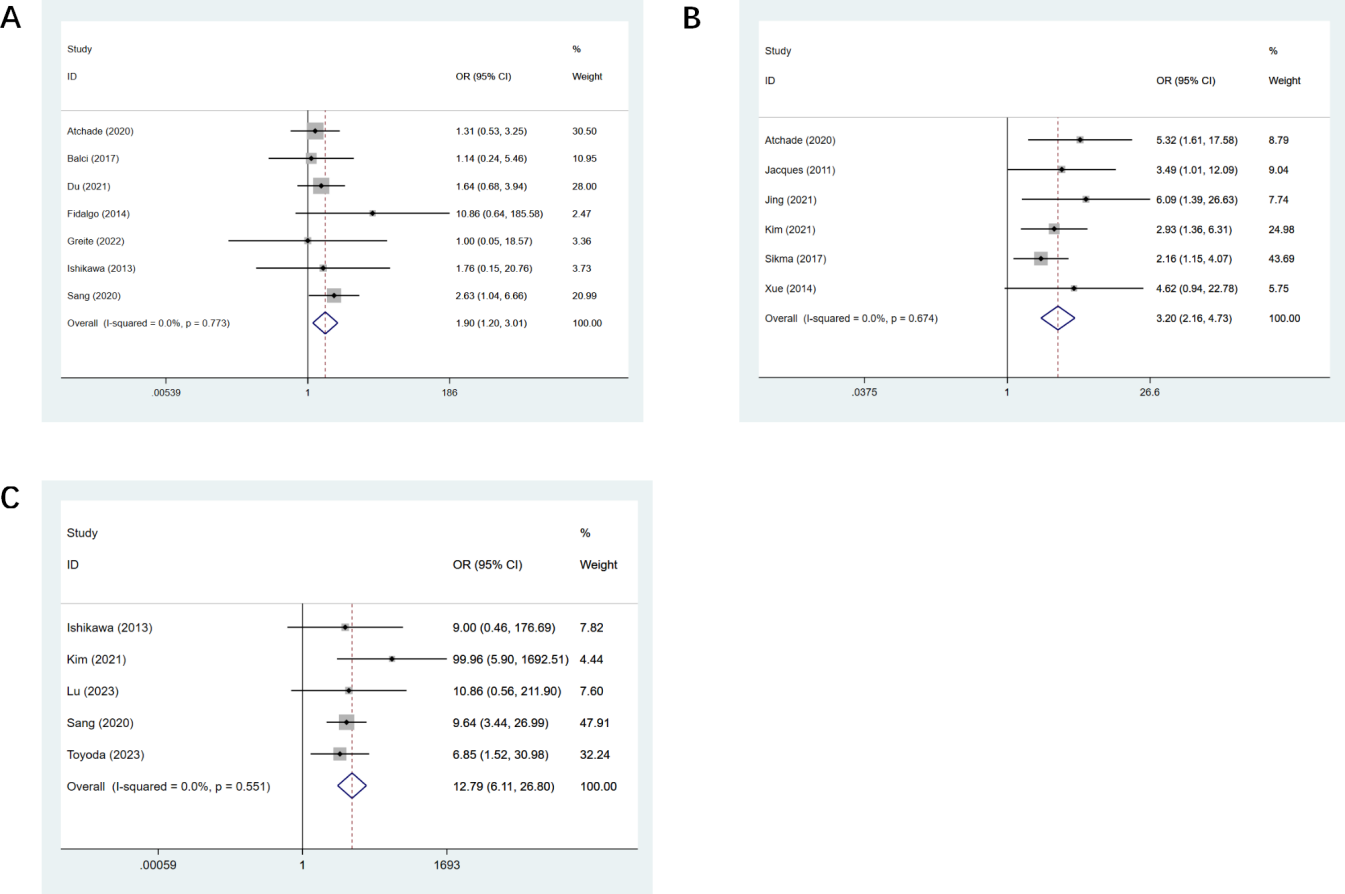
Forest plot showing the results for postoperative univariate analysis. (A) Post-op ECMO; (B) sepsis; (C) dialysis. Atchade et al., 2020; Balci et al., 2017; Fidalgo et al., 2014; Jing et al., 2021; Kim et al., 2021; Lu et al., 2023; Sang et al., 2021; Greite et al., 2022; Ishikawa, Griesdale & Lohser, 2014; Jacques et al., 2012; Rocha et al., 2005; Chan et al., 2023; Du et al., 2021; Toyoda et al., 2023; Sikma et al., 2017; Liu et al., 2021; Xue et al., 2014; Xue et al., 2014; Zhang Shuai & Jingqing, 2020.

Seven studies mentioned post-op ECMO (Atchade et al., 2020; Balci et al., 2017; Du et al., 2021; Fidalgo et al., 2014; Greite et al., 2022; Ishikawa, Griesdale & Lohser, 2014; Sang et al., 2021), and a statistical difference was observed (OR, 1.90; CI [1.20–3.01], Fig. 4A). Six studies mentioned sepsis (Atchade et al., 2020; Jacques et al., 2012; Jing et al., 2021; Kim et al., 2021; Sikma et al., 2017; Xue et al., 2014), and the observed difference was of statistical significance (OR, 3.20; CI [2.16–4.73], Fig. 4B). Five studies mentioned dialysis (Ishikawa, Griesdale & Lohser, 2014; Kim et al., 2021; Lu et al., 2023; Sang et al., 2021; Toyoda et al., 2023), and we observed a statistical difference (OR, 12.79; CI [6.11–26.8], Fig. 4C). Four studies mentioned the duration of ECMO support (Atchade et al., 2020; Lu et al., 2023; Sang et al., 2021; Toyoda et al., 2023), and we observed a statistical difference (OR, 0.36; CI [0.16–0.57]). The duration of mechanical ventilation was mentioned in 12 studies (Atchade et al., 2020; Balci et al., 2017; Du et al., 2021; Ishikawa, Griesdale & Lohser, 2014; Jing et al., 2021; Kim et al., 2021; Liu et al., 2021; Lu et al., 2023; Sang et al., 2021; Toyoda et al., 2023; Xue et al., 2014; Zhang Shuai & Jingqing, 2020), and the difference observed was of statistical significance (OR, 0.47; CI [0.20–0.74]). All results of postoperative univariate analysis are described in Table 3.

### Multivariate meta-analysis

The results of multivariate analysis were pooled and analyzed, and double LT was RFs for AKI after LT, as depicted in Table 4.

## Discussion

This study is the first meta-analysis to explore the possible RFs for AKI after LT. We collected eligible studies published from January 2005 to June 2023 and analyzed the RFs in each study. The results revealed that PF, hypertension, pre-op MV, pre-op ECMO, double LT, CPB support, cardiovascular events, intra-op hypotension, post-op ECMO, sepsis, dialysis may be RFs for AKI after LT, aiming to provide an evidence-based basis for clinically improving the quality of life and reducing postoperative AKI in patients on LT.

AKI refers to a group of clinical syndromes, mainly the renal ischemia-reperfusion injury caused by intra-op blood loss, with rapid increase of serum creatinine (SCr). This condition manifests as azotemia, systemic symptoms, and water-electrolyte and acid–base imbalance, accompanied by oliguria or anuria. Studies have shown that 1/3 of AKI cases occurs in the perioperative period, and it accounts for 18% to 47% of the total number of acquired AKI (Hermansen et al., 2021; Liu et al., 2016; Ronco, Bellomo & Kellum, 2019). Early prediction and treatment of AKI are important to reduce mortality. Hence, identifying individuals who are at an elevated risk of AKI and preventing AKI are crucial. The prediction of AKI is based on Improving Global Outcomes (KDIGO) 2012 guidelines (Khwaja, 2012). However, because of the exponential relationship between serum creatinine and glomerular filtration rate (GFR), creatinine is a late marker for the decline of GFR, and many nephrons may have been lost before serum creatinine rises. Other confounding factors may also further delay or obscure the diagnosis of AKI, especially in more severe cases. For example, fluid overload could dilute serum creatinine, and creatinine production is reduced in patients with sepsis or sarcopenia, possibly leading to false-negative results (Schetz & Schortgen, 2017). Therefore, this study used a systematic review and meta-analysis to explore more prospective RFs for AKI after LT.

This study revealed that PF may be an RF for AKI after LT through the univariate analysis. This suggested that the morbidity rate of AKI in LT recipient with PF was significantly increased. Inflammatory factors produced by lung lesions may activate immune responses *via* the blood system, causing renal autoimmune diseases such as glomerulonephritis (Steele & Schwartz, 2013; White & Mantovani, 2013). Dyspnea is the most common symptom of PF. Long-term hypoxia produces adaptive hypoxia-inducible factors (HIFs) and hypercapnia (Chapman et al., 2021; Higgins et al., 2007; Kimura et al., 2008), which may lead to renal tubulointerstitial damage, renal insufficiency and even uremia.

The morbidity rate of AKI after operation in LT patients with pre-op hypertension or intra-op hypotension was significantly increased showed by the univariate analysis. This might be due to glomerular atherosclerosis caused by persistent uncontrolled hypertension (Denic et al., 2018). Intra-op renal hypoperfusion aggravates renal ischemia, which affects the excretory function of the kidney and induces the loss of control of water and sodium metabolism as well as acid–base balance. Patients with hypertension are prone to developing thrombosis, especially in the renal artery, leading to ischemic necrosis of renal tissues. If vasoactive drugs and anticoagulants are used during the operation, damage to the kidney may be caused (Charles, Triscott & Dobbs, 2017; Roberts et al., 2015).

This study also indicated that pre-op mechanical ventilation, a very high risk of the renal angina construct (Basu et al., 2014; Matsuura et al., 2018), may be an RF for AKI after LT by the univariate analysis, and the potential mechanisms may involve hemodynamic factors, selective renal vasoconstriction activated by MV-induced sympathetic nerve stimulation, and inflammatory response to ventilator-induced barotrauma, eventually resulting in renal hypoperfusion (Husain-Syed, Slutsky & Ronco, 2016; Liu & Matthay, 2008; Van den Akker, Egal & Groeneveld, 2013).

In this study, pre-op or post-op ECMO was found to be an RF for AKI after LT through the univariate analysis. This was because the patients had prior health problems such as severe hypoxemia, low cardiac output, neurohormonal imbalance, high intrathoracic pressure, hypercapnia and hemodynamic instability (Prodhan et al., 2014; Ramanathan et al., 2017; Schmidt et al., 2014). As an invasive procedure, ECMO may cause problems such as elevated intra-abdominal pressure and congestion in renal, which impaired renal blood flow as well as cardiorenal syndrome. Additionally, hemolysis is a common complication of ECMO (Pan et al., 2016). When the concentration of free hemoglobin exceeds 130 mg/L, hemoglobinuria will occur. The hemolysate will damage renal tubular cells, causing necrosis, hemoglobin deposition in renal tubules, and weak peripheral circulation. The frequent ECMO is related to the critical condition of the patients. It is impossible for us to clarify whether AKI is caused by ECMO or the critical condition that requires ECMO. Therefore, it can only be concluded that ECMO is related to AKI and may be one of RFs.

This study showed that double LT may be an RF for AKI after LT through the univariate and multivariate analyses. When analyzing possible RFs for AKI, we find a statistical difference between double LT (OR, 1.91; CI [1.45–2.53]) and single LT (OR, 0.52; CI [0.42–0.64]), which suggests that double LT is a RF for AKI, while single LT is a protective factor. To eliminate the interference caused by the older age of patients on single LT, we re-analyzed the studies (Balci et al., 2017; Greite et al., 2022; Jacques et al., 2012; Scaravilli et al., 2022; Sikma et al., 2017) in which at least 84% of the patients were not older than 65 years old (one of the indications for double LT), that is, (Mean + SD) ≤ 65. The same results were observed (double LT (OR, 2.21; CI [1.41–3.47]), *p* = 0.001; single LT (OR, 0.42; CI [0.23–0.75]), *p* = 0.003). Hence, a double lung transplant may be associated with AKI. In double LT, the medical technologies required in lung resection and implantation are more complex, and the operation time and surgical trauma are significantly greater than those of single LT, increasing the risk of bleeding and ischemic injury (Huang et al., 2023).

This study also indicated that CPB surgery may be an RF for AKI after LT by the univariate analysis. CPB surgery requires the patient’s blood to be taken out of the body and circulated through a machine. Prolonged CPB leads to renal ischemia and hypoxia. Furthermore, the patients may have hypothermia, and the perfusion pressure of the kidney will be reduced (Huang et al., 2023; Karthik et al., 2004; Patel et al., 2002; Stamou et al., 2002).

This study also indicated that intra-op cardiovascular events may be an RF for AKI after LT by the univariate analysis. LT is a major operation requiring support such as general anesthesia and cardiopulmonary bypass, which leads to a stress state in patients and increased release of vasoactive substances such as adrenaline and norepinephrine in the body (Hoeper et al., 2019; Kolaitis, 2023; Sultan et al., 2018). Severe hypotension can lead to decreased renal perfusion pressure. Thrombus, especially deep venous thrombosis of the lower extremities, falls off and enters the kidney with the blood (Daniels et al., 2012; Mazer et al., 2023; Ng et al., 2016; Wetz et al., 2015).

We also found that post-op sepsis may be an independent RF for AKI after LT through the univariate analysis. Sepsis should be defined as life-threatening organ dysfunction caused by a dysregulated host response to infection. The following parameters can be used to define sepsis: temperature <36 °C or >38 °C, heart rate >90 bpm, white blood count >12 or <4 × 10^6^/mL, respiratory rate >20 or hyperglycemia without diabetes (Singer et al., 2016). The most common organ dysfunction during sepsis is AKI. Several lines of evidence suggest that renal tubular epithelial injury is a prominent feature of sepsis-associated AKI (Alobaidi et al., 2015; Prince, Tafur & White, 2019). Due to the large surgical wound surface, large amount of bleeding, and the need to use immunosuppressants after the operation, patients’ immunity decreases, bacteria multiply in the body and release toxins, triggering an inflammatory response that leads to the development of sepsis (Inagaki, Weinberg & Kaul, 2023; Onyearugbulem et al., 2018; Takeda et al., 2016). AKI caused by sepsis is mainly due to systemic damage (Poston & Koyner, 2019). For the kidney, sepsis can lead to changes in microcirculation, endothelial cell inflammatory injury and interstitial edema, hypoperfusion caused by hypovolemia and hypotension, ischemia and reperfusion, exogenous nephrotoxin, resulting in oliguria or even anuria. Besides, toxins cannot be cleared due to insufficient renal perfusion (Peerapornratana et al., 2019; Yue et al., 2022).

Dialysis may also be an RF for AKI after LT by the univariate analysis. Dialysis is suitable for patients with severe renal failure, whose basic renal function is poor. In dialysis, hemodynamic changes, tissue hypoperfusion, insufficient oxygen supply and other factors may lead to acute tubular necrosis. Thus, thrombus may occur to block renal tubules or renal interstitial blood vessels, which may lead to AKI. If the dialyzer, filter or tubes used for dialysis are incompatible with the patient, allergic reaction or inflammatory reaction may be caused (Gaudry et al., 2022; Gaudry et al., 2021; Li et al., 2021). However, indications of dialysis include life-threatening or severe complications of uremia and persistent oliguria or anuria (Kotloff, 2013; Teixeira, Neyra & Tolwani, 2023), which means frequent dialysis is related to the critical renal condition of the patients. Thus it can only be concluded that there is an association between dialysis and postoperative AKI.

Lung allocation score (LAS) is a scoring method used to measure the medical urgency status of people waiting for LT with higher scores representing higher medical urgency status. The reason why people with high LAS score are prone to AKI may be related to the pathophysiological mechanism of AKI, resulting in severe hypoxia and metabolic disorders in the body. This state may lead to a decrease in the blood supply to the kidney and a decrease in glomerular filtration rate, thereby causing AKI.

The longer the duration of LT, the greater the impact on the physiological state of the patients, and this may lead to the reduction of renal blood perfusion, thus causing AKI.

The greater the amount of RBC transfusion during the operation, the more likely the patient will have AKI after the operation. Transfusion of a large amount of RBC may cause transfusion-associated acute kidney injury (TAI), which is a common complication of blood transfusion; RBCs may produce some harmful substances during storage, such as potassium and ammonia, which may cause kidney damage during transfusion. The transfused RBC may cause an immune response in the body, leading to AKI resulting from an inflammatory response and the deposition of antigen-antibody complexes (Banerjee et al., 2003; Luhe et al., 2008). However, the use of blood products can also be attributable to hemodynamic instability, for instance, acute hemorrhage during surgery, chronic anemia caused by compromised red blood cell production and metabolism due to specific diseases (uremia) (Carson, Triulzi & Ness, 2017; Chan et al., 2023). It remains unclear whether kidney damage is caused by hemodynamic instability or blood products. It can only be concluded that there is an association between blood transfusion and postoperative AKI.

We have discussed in detail the causes of postoperative AKI caused by duration of ECMO support and MV through direct or indirect factors, and therefore, we will not repeat it here. The diagnostic criteria for AKI across the included studies encompass KDIGO (Improving Global Outcomes Acute Kidney Injury Criteria) (Khwaja, 2012), AKIN (Acute Kidney Injury Net classification) (Mehta et al., 2007), and RIFLE (The risk, injury, failure, loss, endstage RIFLE renal disease criteria) (Bellomo et al., 2004), which have characteristics of the times. The three criteria mainly differed in the staging, while their criteria for diagnosing AKI are similar (Table 1). The focus of this study is on whether AKI occurs after surgery. Consequently, the consistency would not be affected by their difference in staging. Additionally, the difference in the diagnostic criteria for AKI stems from the update of diagnostic cognition. These criteria were developed based on the scientific evidence and clinical experience available at the time, and thus they reflected the level of knowledge, technical capabilities, and research focus in the medical field and represented the best medical practices at the time. Therefore, the difference in the definitions of AKI in numerous studies across time spans is acceptable.

Given the nephrotoxicity of drugs, we analyzed specific medications, including the antiviral drug ganciclovir, the immunosuppressant basiliximab, and antibiotics such as cyclosporine, tacrolimus, and amphotericin Vitamin B. Unfortunately, these variables were excluded due to a lack of statistical significance or publication bias caused by the small number of studies. Possibly because the concentration of each drug was strictly controlled within the safe range, so no detectable renal toxicity was observed.

This study focuses on AKI due to its high occurrence rate and poor prognosis in clinical practice. A mild AKI is generally not associated with any adverse effects. For instance, patients on acute kidney injury-renal replacement therapy (AKI-RRT) had a lower survival rate compared with those on the acute kidney injury-no renal replacement therapy (AKI-no RRT) (Viera, 2008), suggesting that our study focus should be shifted to AKI-RRT and other severe stage AKI. However, near half of the included studies did not classify AKI (Atchade et al., 2020; Balci et al., 2017; Du et al., 2021; Greite et al., 2022; Ishikawa, Griesdale & Lohser, 2014; Jacques et al., 2012; Kim et al., 2021; Liu et al., 2021). Although other studies performed grouping, their grouping methods are different. Therefore, it is difficult for us to conduct subgroup analysis based on enough studies. Thereby, we can only roughly calculate the occurrence rate of AKI of all severities. In our future studies, we will place more focus on the grading of AKI to improve the methodological design. Some other limitations to our analysis should be mentioned. First, the number of studies included is small, and there may be a selection bias. However, we have comprehensively searched relevant studies on sepsis, to reduce publication bias caused by missing studies with negative results. Secondly, each included study may have sampling bias. Because patients with preoperative renal insufficiency may be excluded when they determine candidates for LT, this would greatly reduce the association of renal function markers with preoperative renal disease and postoperative AKI. Additionally, there was no information on the time of occurrence of AKI and postoperative complications in the included studies. Hence, it is challenging to fully elucidate the causal or temporal relationships related to AKI. Besides, the heterogeneity of occurrence rate of AKI after LT was large. To explore the source of heterogeneity, subgroup analysis was conducted by subgrouping the studies by country, continent or level of national development, study type or diagnostic criteria, the results reveal that the heterogeneity may be attributable to case-control studies (Supplemental Information 3), largely because such studies are retrospective, namely, to identify factors that may be related to a disease from established cases, so the occurrence rate is not objective in case-control studies compared to cohort studies. Finally, when conducting multivariate analysis, we analyzed studies that used HR (Atchade et al., 2020; Balci et al., 2017; Du et al., 2021; Fidalgo et al., 2014; Greite et al., 2022; Ishikawa, Griesdale & Lohser, 2014; Jacques et al., 2012; Kim et al., 2021; Liu et al., 2021; Lu et al., 2023; Rocha et al., 2005; Sang et al., 2021; Scaravilli et al., 2022; Xue et al., 2014; Zhang Shuai & Jingqing, 2020) and OR (Chan et al., 2023; Jing et al., 2021; Kim et al., 2021; Toyoda et al., 2023), respectively, so the number of studies on each variable is reduced, which possibly leading to a certain bias in the results.

## Conclusions

Based on the existing evidence, pulmonary fibrosis, hypertension, pre-op mechanical ventilation, pre-op ECMO, double lung transplantation, cardiopulmonary bypass support, cardiovascular events, intra-op hypotension, post-op ECMO, sepsis and dialysis are RFs for AKI after LT. Clinical professionals can use these indicators to implement early detection, diagnosis and treatment of patients with AKI after LT, to improve the quality of life of these patients.

## Supplemental Information

10.7717/peerj.18364/supp-1 Supplemental Information 1 The specific retrieval strategies

10.7717/peerj.18364/supp-2 Supplemental Information 2 Statistical diagram of the incidence of acute kidney injury in lung transplantation (A) The forest plot of the incidence of acute kidney injury in lung transplantation. (B) The Meta-analysis estimates of the incidence of acute kidney injury in lung transplantation. (C) The Egger’s publication bias plot of the incidence of acute kidney injury in lung transplantation.

10.7717/peerj.18364/supp-3 Supplemental Information 3 The type of case-control study is the source of significant heterogeneity

10.7717/peerj.18364/supp-4 Supplemental Information 4 The original data of multivariate analysis

10.7717/peerj.18364/supp-5 Supplemental Information 5 PRISMA checklist

## Additional Information and Declarations

### Data Availability

The following information was supplied regarding data availability: This is a systematic review/meta-analysis.
