# Supplementary material for: Occurrence rate and risk factors for acute kidney injury after lung transplantation: a systematic review and meta-analysis
Source: PeerJ. 2025 Feb 21;13:e18364. doi: 10.7717/peerj.18364 (PMC11849521; doi:10.7717/peerj.18364)
Supplement: Supplemental Information 1 [file peerj-13-18364-s001.docx]

Retrieval Platform:CNKI

Condition: (Subject: "Lung Transplantation"+"Single Lung Transplantation"+"Unilateral Lung Transplantation"+"In Situ Lung Transplantation"+"Double Lung Transplantation"+"Allogeneic Lung Transplantation"+"Lung Transplantation" (exact) OR (Title: "Lung Transplantation"+"Single Lung Transplantation"+"Unilateral Lung Transplantation"+"In Situ Lung Transplantation"+"Double Lung Transplantation"+"Allogeneic Lung Transplantation"+"Lung Transplantation" (exact)) AND (Subject: "Acute Kidney Injury"+"Acute Interstitial Nephritis"+"Acute Renal Insufficiency"+"Acute Kidney Failure"+"Acute Kidney Injury"+"Acute Kidney Injury"+"Acute Kidney Injury"+"Acute Kidney Injury"+"Renal tubular necrosis"+"acute renal insufficiency"+"acute renal failure"+"acute renal damage"+"renal function (precise)" OR (article excerpt: "acute renal injury") +Acute interstitial nephritis "+" acute renal insufficiency "+" acute renal failure "+" acute renal injury "+" acute renal injury "+" acute tubular necrosis "+" acute renal insufficiency "+" acute renal failure "+" acute renal injury "+" renal function ") AND ((Theme:" Risk factors "+" factors "+" hidden dangers "+" hidden dangers "(exact)) OR (Excerpt:" Risk factors "+" factors "+" hidden dangers "+" hidden dangers ")

Scope: LiteratureData run:2023-08-22 21:20:30

Retrieval Platform:Pubmed

Search number Query Sort By Filters Search Details Results Time

1 (("Lung Transplantation"[Mesh]) OR (lung transplantation[Title/Abstract] OR grafting, lung[Title/Abstract] OR graftings, lung[Title/Abstract] OR lung allograft[Title/Abstract] OR lung allotransplantation[Title/Abstract] OR lung grafting[Title/Abstract] OR lung graftings[Title/Abstract] OR lung orthotopic transplantation[Title/Abstract] OR lung tissue transplantation[Title/Abstract] OR lung transplant[Title/Abstract] OR lung transplantations[Title/Abstract] OR pulmonary transplantation[Title/Abstract] OR transplantation, lung[Title/Abstract] OR transplantations, lung[Title/Abstract])) AND (("acute kidney injury"[Mesh]) OR (acute kidney injury[Title/Abstract] OR acute kidney failure[Title/Abstract] OR acute kidney failures[Title/Abstract] OR acute kidney injuries[Title/Abstract] OR acute kidney insufficiencies[Title/Abstract] OR acute kidney insufficiency[Title/Abstract] OR acute renal failure[Title/Abstract] OR acute renal failures[Title/Abstract] OR acute renal injuries[Title/Abstract] OR acute renal injury[Title/Abstract] OR acute renal insufficiencies[Title/Abstract] OR acute renal insufficiency[Title/Abstract] OR aki[Title/Abstract] OR kidney acute failure[Title/Abstract] OR kidney failure, acute[Title/Abstract] OR kidney failures, acute[Title/Abstract] OR kidney injuries, acute[Title/Abstract] OR kidney injury, acute[Title/Abstract] OR kidney insufficiencies, acute[Title/Abstract] OR kidney insufficiency, acute[Title/Abstract] OR renal failure, acute[Title/Abstract] OR renal failures, acute[Title/Abstract] OR renal injuries, acute[Title/Abstract] OR renal injury, acute[Title/Abstract] OR renal insufficiencies, acute[Title/Abstract] OR renal insufficiency, acute[Title/Abstract])) AND (("risk factors"[Mesh]) OR (risk factors[Title/Abstract] OR correlates, health[Title/Abstract] OR factor, risk[Title/Abstract] OR factor, social risk[Title/Abstract] OR factors, social risk[Title/Abstract] OR health correlates[Title/Abstract] OR population at risk[Title/Abstract] OR populations at risk[Title/Abstract] OR relative risk[Title/Abstract] OR risk factor[Title/Abstract] OR risk factor score[Title/Abstract] OR risk factor scores[Title/Abstract] OR risk factor, social[Title/Abstract] OR risk factors, social[Title/Abstract] OR risk score[Title/Abstract] OR risk scores[Title/Abstract] OR score, risk[Title/Abstract] OR score, risk factor[Title/Abstract] OR social risk factor[Title/Abstract] OR social risk factors[Title/Abstract] OR social risk factors[Title/Abstract])) ("Lung Transplantation"[MeSH Terms] OR ("Lung Transplantation"[Title/Abstract] OR "grafting lung"[Title/Abstract] OR (("graft s"[All Fields] OR "grafted"[All Fields] OR "graftings"[All Fields] OR "transplantation"[MeSH Subheading] OR "transplantation"[All Fields] OR "grafting"[All Fields] OR "transplantation"[MeSH Terms] OR "grafts"[All Fields] OR "transplants"[MeSH Terms] OR "transplants"[All Fields] OR "graft"[All Fields]) AND "lung"[Title/Abstract]) OR "lung allograft"[Title/Abstract] OR "lung allotransplantation"[Title/Abstract] OR "lung grafting"[Title/Abstract] OR (("lung"[MeSH Terms] OR "lung"[All Fields]) AND "graftings"[Title/Abstract]) OR (("lung"[MeSH Terms] OR "lung"[All Fields]) AND "orthotopic transplantation"[Title/Abstract]) OR (("lung"[MeSH Terms] OR "lung"[All Fields]) AND "tissue transplantation"[Title/Abstract]) OR "lung transplant"[Title/Abstract] OR "lung transplantations"[Title/Abstract] OR "pulmonary transplantation"[Title/Abstract] OR "transplantation lung"[Title/Abstract] OR "transplantations lung"[Title/Abstract])) AND ("acute kidney injury"[MeSH Terms] OR ("acute kidney injury"[Title/Abstract] OR "acute kidney failure"[Title/Abstract] OR "acute kidney failures"[Title/Abstract] OR "acute kidney injuries"[Title/Abstract] OR "acute kidney insufficiencies"[Title/Abstract] OR "acute kidney insufficiency"[Title/Abstract] OR "acute renal failure"[Title/Abstract] OR "acute renal failures"[Title/Abstract] OR "acute renal injuries"[Title/Abstract] OR "acute renal injury"[Title/Abstract] OR "acute renal insufficiencies"[Title/Abstract] OR "acute renal insufficiency"[Title/Abstract] OR "aki"[Title/Abstract] OR (("kidney"[MeSH Terms] OR "kidney"[All Fields] OR "kidneys"[All Fields] OR "kidney s"[All Fields]) AND "acute failure"[Title/Abstract]) OR "kidney failure acute"[Title/Abstract] OR (("kidney"[MeSH Terms] OR "kidney"[All Fields] OR "kidneys"[All Fields] OR "kidney s"[All Fields]) AND "failures acute"[Title/Abstract]) OR "kidney injuries acute"[Title/Abstract] OR "kidney injury acute"[Title/Abstract] OR (("renal insufficiency"[MeSH Terms] OR ("renal"[All Fields] AND "insufficiency"[All Fields]) OR "renal insufficiency"[All Fields] OR ("kidney"[All Fields] AND "insufficiencies"[All Fields]) OR "kidney insufficiencies"[All Fields]) AND "acute"[Title/Abstract]) OR (("kidney"[MeSH Terms] OR "kidney"[All Fields] OR "kidneys"[All Fields] OR "kidney s"[All Fields]) AND "insufficiency acute"[Title/Abstract]) OR "renal failure acute"[Title/Abstract] OR (("renal"[All Fields] OR "renals"[All Fields]) AND "failures acute"[Title/Abstract]) OR "renal injuries acute"[Title/Abstract] OR "renal injury acute"[Title/Abstract] OR (("renal insufficiency"[MeSH Terms] OR ("renal"[All Fields] AND "insufficiency"[All Fields]) OR "renal insufficiency"[All Fields] OR ("renal"[All Fields] AND "insufficiencies"[All Fields]) OR "renal insufficiencies"[All Fields]) AND "acute"[Title/Abstract]) OR "renal insufficiency acute"[Title/Abstract])) AND ("risk factors"[MeSH Terms] OR ("risk factors"[Title/Abstract] OR "correlates health"[Title/Abstract] OR "factor risk"[Title/Abstract] OR (("factor"[All Fields] OR "factor s"[All Fields] OR "factors"[All Fields]) AND "social risk"[Title/Abstract]) OR "factors social risk"[Title/Abstract] OR "health correlates"[Title/Abstract] OR "population at risk"[Title/Abstract] OR "populations at risk"[Title/Abstract] OR "relative risk"[Title/Abstract] OR "risk factor"[Title/Abstract] OR "risk factor score"[Title/Abstract] OR "risk factor scores"[Title/Abstract] OR "risk factor social"[Title/Abstract] OR "risk factors social"[Title/Abstract] OR "risk score"[Title/Abstract] OR "risk scores"[Title/Abstract] OR "score risk"[Title/Abstract] OR "score risk factor"[Title/Abstract] OR "social risk factor"[Title/Abstract] OR "social risk factors"[Title/Abstract] OR "social risk factors"[Title/Abstract])) 113 9:49:26

Retrieval Platform:Embase

Session Results

.......................................................

No. Query Results Results Date

#1. ('lung transplantation'/exp OR 'lung 199 22 Aug 2023

transplantation' OR 'lung

transplantation':ti,ab,kw OR 'grafting,

lung':ti,ab,kw OR 'graftings, lung':ti,ab,kw OR

'lung allograft':ti,ab,kw OR 'lung

allotransplantation':ti,ab,kw OR 'lung

grafting':ti,ab,kw OR 'lung graftings':ti,ab,kw

OR 'lung orthotopic transplantation':ti,ab,kw OR

'lung tissue transplantation':ti,ab,kw OR 'lung

transplant':ti,ab,kw OR 'lung

transplantations':ti,ab,kw OR 'pulmonary

transplantation':ti,ab,kw OR 'transplantation,

lung':ti,ab,kw OR 'transplantations,

lung':ti,ab,kw) AND ('acute kidney injury'/exp OR

'acute kidney injury' OR 'acute kidney

injury':ti,ab,kw OR 'acute kidney

failure':ti,ab,kw OR 'acute kidney

failures':ti,ab,kw OR 'acute kidney

injuries':ti,ab,kw OR 'acute kidney

insufficiencies':ti,ab,kw OR 'acute kidney

insufficiency':ti,ab,kw OR 'acute renal

failure':ti,ab,kw OR 'acute renal

failures':ti,ab,kw OR 'acute renal

injuries':ti,ab,kw OR 'acute renal

injury':ti,ab,kw OR 'acute renal

insufficiencies':ti,ab,kw OR 'acute renal

insufficiency':ti,ab,kw OR 'aki':ti,ab,kw OR

'kidney acute failure':ti,ab,kw OR 'kidney

failure, acute':ti,ab,kw OR 'kidney failures,

acute':ti,ab,kw OR 'kidney injuries,

acute':ti,ab,kw OR 'kidney injury,

acute':ti,ab,kw OR 'kidney insufficiencies,

acute':ti,ab,kw OR 'kidney insufficiency,

acute':ti,ab,kw OR 'renal failure,

acute':ti,ab,kw OR 'renal failures,

acute':ti,ab,kw OR 'renal injuries,

acute':ti,ab,kw OR 'renal injury, acute':ti,ab,kw

OR 'renal insufficiencies, acute':ti,ab,kw OR

'renal insufficiency, acute':ti,ab,kw) AND ('risk

factors'/exp OR 'risk factors' OR 'risk

factors':ti,ab,kw OR 'correlates,

health':ti,ab,kw OR 'factor, risk':ti,ab,kw OR

'factor, social risk':ti,ab,kw OR 'factors,

social risk':ti,ab,kw OR 'health

correlates':ti,ab,kw OR 'population at

risk':ti,ab,kw OR 'populations at risk':ti,ab,kw

OR 'relative risk':ti,ab,kw OR 'risk

factor':ti,ab,kw OR 'risk factor score':ti,ab,kw

OR 'risk factor scores':ti,ab,kw OR 'risk factor,

social':ti,ab,kw OR 'risk factors,

social':ti,ab,kw OR 'risk score':ti,ab,kw OR

'risk scores':ti,ab,kw OR 'score, risk':ti,ab,kw

OR 'score, risk factor':ti,ab,kw OR 'social risk

factor':ti,ab,kw OR 'social risk

factors':ti,ab,kw)

.......................................................

Retrieval Platform:Cochrane

Search Name:

Date Run: 22/08/2023 10:04:48

Comment:

ID Search Hits

#1 MeSH descriptor: [Lung Transplantation] explode all trees 380

#2 (“lung transplantation” or “grafting, lung” or “graftings, lung” or “lung allograft” or “lung allotransplantation” or “lung grafting” or “lung graftings” or “lung orthotopic transplantation” or “lung tissue transplantation” or “lung transplant” or “lung transplantations” or “pulmonary transplantation” or “transplantation, lung” or “transplantations, lung”):ti,ab,kw 1295

#3 MeSH descriptor: [Acute Kidney Injury] explode all trees 2034

#4 (“acute kidney injury” or “acute kidney failure” or “acute kidney failures” or “acute kidney injuries” or “acute kidney insufficiencies” or “acute kidney insufficiency” or “acute renal failure” or “acute renal failures” or “acute renal injuries” or “acute renal injury” or “acute renal insufficiencies” or “acute renal insufficiency” or “aki” or “kidney acute failure” or “kidney failure, acute” or “kidney failures, acute” or “kidney injuries, acute” or “kidney injury, acute” or “kidney insufficiencies, acute” or “kidney insufficiency, acute” or “renal failure, acute” or “renal failures, acute” or “renal injuries, acute” or “renal injury, acute” or “renal insufficiencies, acute” or “renal insufficiency, acute”):ti,ab,kw 7002

#5 MeSH descriptor: [Risk Factors] explode all trees 33162

#6 (“risk factors” or “correlates, health” or “factor, risk” or “factor, social risk” or “factors, social risk” or “health correlates” or “population at risk” or “populations at risk” or “relative risk” or “risk factor” or “risk factor score” or “risk factor scores” or “risk factor, social” or “risk factors, social” or “risk score” or “risk scores” or “score, risk” or “score, risk factor” or “social risk factor” or “social risk factors” or “social risk factors”):ti,ab,kw 93797

#7 (#1 or #2) and (#3 or #4) and (#5 or #6) 7

Retrieval Platform:WOS

# Web of Science Search Strategy (v0.1)

# Database: All Databases

# Entitlements:

- WOS: 1900 to 2023

- BIOSIS: 2002 to 2014

- CSCD: 1989 to 2023

- DIIDW: 1966 to 2022

- KJD: 1980 to 2023

- MEDLINE: 1950 to 2023

- PPRN: 1991 to 2023

- PQDT: 1637 to 2023

- SCIELO: 2002 to 2023

# Searches:

1: TS=(“lung transplantation” or “grafting, lung” or “graftings, lung” or “lung allograft” or “lung allotransplantation” or “lung grafting” or “lung graftings” or “lung orthotopic transplantation” or “lung tissue transplantation” or “lung transplant” or “lung transplantations” or “pulmonary transplantation” or “transplantation, lung” or “transplantations, lung”) AND TS=(“acute kidney injury” or “acute kidney failure” or “acute kidney failures” or “acute kidney injuries” or “acute kidney insufficiencies” or “acute kidney insufficiency” or “acute renal failure” or “acute renal failures” or “acute renal injuries” or “acute renal injury” or “acute renal insufficiencies” or “acute renal insufficiency” or “aki” or “kidney acute failure” or “kidney failure, acute” or “kidney failures, acute” or “kidney injuries, acute” or “kidney injury, acute” or “kidney insufficiencies, acute” or “kidney insufficiency, acute” or “renal failure, acute” or “renal failures, acute” or “renal injuries, acute” or “renal injury, acute” or “renal insufficiencies, acute” or “renal insufficiency, acute”) AND TS=(“risk factors” or “correlates, health” or “factor, risk” or “factor, social risk” or “factors, social risk” or “health correlates” or “population at risk” or “populations at risk” or “relative risk” or “risk factor” or “risk factor score” or “risk factor scores” or “risk factor, social” or “risk factors, social” or “risk score” or “risk scores” or “score, risk” or “score, risk factor” or “social risk factor” or “social risk factors” or “social risk factors”) and Preprint Citation Index (Exclude – Database) Date Run: Mon Aug 22 2023 22:08:54 GMT+0800 (China Standard Time) Results: 130
