# Supplementary figures and images for: Occurrence rate and risk factors for acute kidney injury after lung transplantation: a systematic review and meta-analysis
Source: PeerJ. 2025 Feb 21;13:e18364. doi: 10.7717/peerj.18364 (PMC11849521; doi:10.7717/peerj.18364)

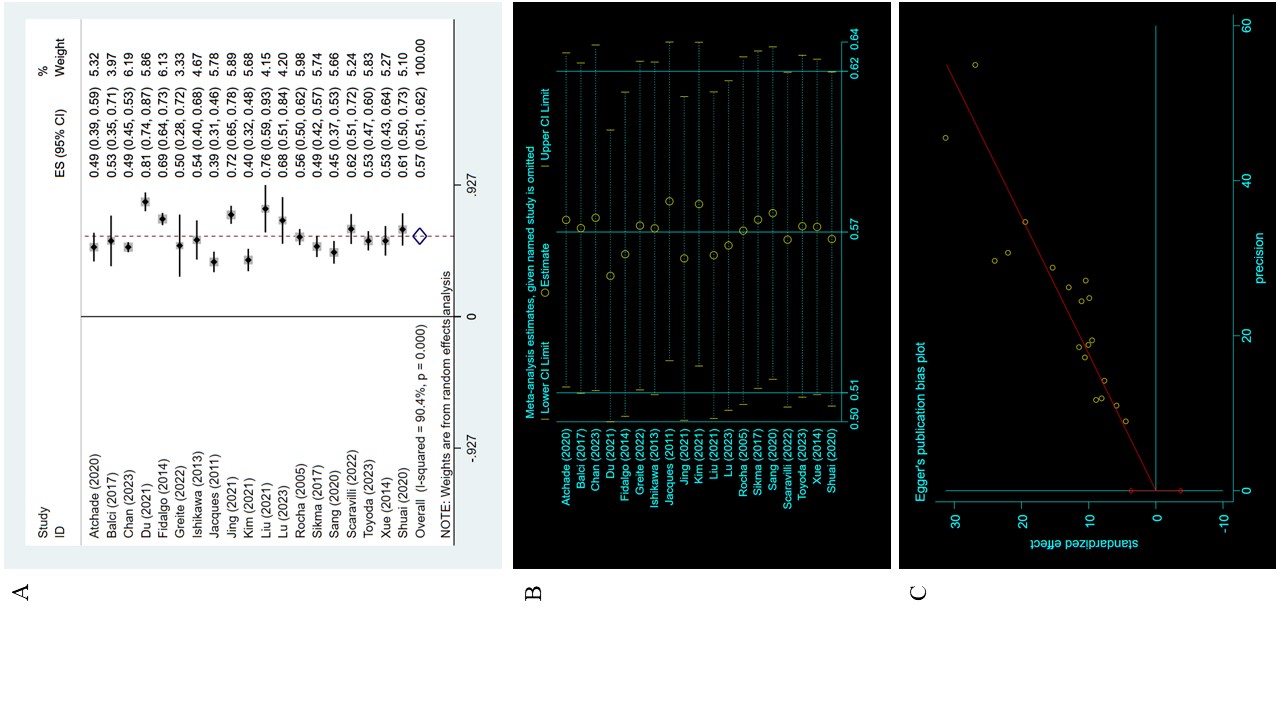

Supplement: Supplemental Information 2 — (A) The forest plot of the incidence of acute kidney injury in lung transplantation. (B) The Meta-analysis estimates of the incidence of acute kidney injury in lung transplantation. (C) The Egger’s publication bias plot of the incidence of acute kidney injury in lung transplantation. [file peerj-13-18364-s002.jpg]

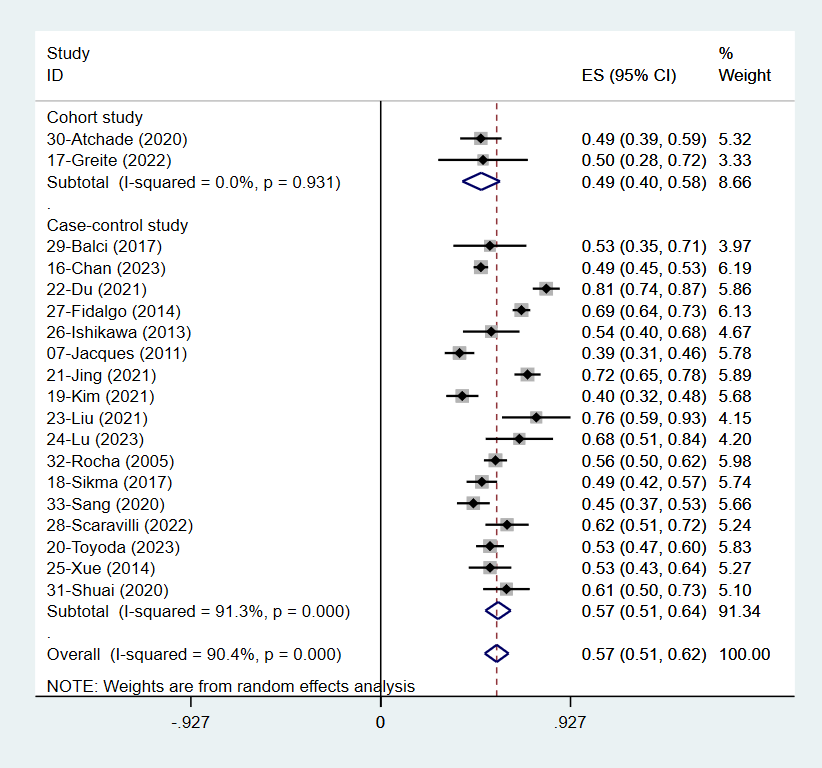

Supplement: Supplemental Information 3 [file peerj-13-18364-s003.png]
